# A nurse-led conceptual model to inform patient-centred, type 2 diabetes mellitus management in public clinical settings

**DOI:** 10.1177/17449871211021137

**Published:** 2021-12-21

**Authors:** Stella Foluke Bosun-Arije, Candidus Chibuzor Nwakasi, Mandu Ekpenyong, Laura Serrant, Temitope Esther Sunday-Abel, Jonathan Ling

**Affiliations:** Senior Lecturer, Department of Nursing, Manchester Metropolitan University, UK; Assistant Professor, Department of Health Policy and Management, Providence College, USA; Research Fellow, Department of Nursing, Manchester Metropolitan University, UK; Professor, Department of Nursing, Manchester Metropolitan University, UK; Assistant Chief Nursing Officer, Federal Medical Centre, Lagos, Nigeria; Professor, Faculty of Health Sciences and Wellbeing, University of Sunderland, UK

**Keywords:** health system, hospitals, nurses, patient-centred care, public, type 2 diabetes mellitus

## Abstract

**Background:**

Globally, there is an increased need to provide patient-centred care for people diagnosed with type 2 diabetes mellitus. In Nigeria, a poorly financed health system has worsened the difficulties associated with managing type 2 diabetes mellitus in clinical settings, causing a detrimental effect on patient-centred care.

**Aims:**

We aimed to develop a conceptual model to promote patient-centred type 2 diabetes mellitus care in clinical settings. We explored nurses’ contextual perceptions of clinical practices and operations in light of type 2 diabetes mellitus management across public hospitals in Lagos, Nigeria. Identifying a nurse-led intervention is critical to care optimisation for people diagnosed with type 2 diabetes mellitus.

**Methods:**

We adopted a qualitative approach. Using the constant comparison method and semi-structured questions and interviewed practice nurses, with over one year’s experience and who were working in public hospitals across Lagos, Nigeria. The framework method was used to analyse the data obtained.

**Results:**

Nurses provided insight into four areas of patient-centred type 2 diabetes mellitus management in clinical settings: empowering collaboration; empowering flexibility; empowering approach; and empowering practice. Nurses discussed an empowering pathway through which health settings could provide patient-centred care to individuals diagnosed with type 2 diabetes mellitus. The pathway entailed the integration of macro, meso and micro levels for patient management. Nurses’ accounts have informed the development of a conceptual model for the optimisation of patient care.

**Conclusions:**

The model developed from this research sits within the patient-centred care model of healthcare delivery. The research sits within the patient-centred care model of healthcare delivery. inform patient-centred care, not only in countries with poorly financed healthcare systems, but in developed countries with comparatively better healthcare.

## Introduction

The prevalence of diabetes mellitus (DM) is increasing across the world and, notably, in low and middle-income countries (LMICs). Type 2 diabetes mellitus (T2DM) is the most prevalent type of DM in Nigeria (International Diabetes Federation, 2015), accounting for 95% of DM cases reported in Nigerian hospitals (Chinenye et al., 2012). However, 70–80% of individuals remain undiagnosed in the community (International Diabetes Federation, 2014). Almost 60% of those diagnosed with T2DM had an early onset of complications such as hypertension (Anakwue et al., 2013; Fasanmade et al., 2013) and 90% developed dyslipidaemia (Chinenye et al., 2012). A substantial proportion of patients with T2DM in Nigeria had below intermediate level quality of life, and were physically inactive (Adeniyi et al., 2015). The reported burdens occurred because approximately 70% of Nigerians live in poverty (National Bureau of Statistics, 2015) and there are organisational factors such as poor knowledge and information management that can adversely impact patient care (Bosun-Arije et al., 2020).

The burdens associated with T2DM management in Nigeria are driven by a combination of complex factors such as poverty, lack of health-related knowledge and a fragile healthcare system (National Bureau of Statistics, 2015). For instance, in 2017, only 3.76% of the Nigerian national budget was allocated to the health sector compared to countries such as Sierra Leone (13.42%), South Africa (8.11%) and Malawi (9.65%) (World Bank, 2017).

A systematic review by Bosun-Arije et al. (2019) found six factors influencing T2DM management in Nigerian public hospitals: non-adherence/non-compliance, biosocial, self-care, psychological, as well as cost and drug-related. These factors are detrimental to patient-centredness and optimisation of patient care (Iwuala et al., 2015). Patient-centred care improves patient self-management skills and heightened patient quality of life. Challenges of T2DM management include prolonged hospital stays, high medical costs, microvascular and macrovascular complications (Arogundade, 2013; Olamoyegun et al., 2015; Onakpoya et al., 2010) and a high mortality rate (Chijioke et al., 2010). These burdens threaten patient-centred care and reduce the optimisation of patient empowerment. Structured recommendations are required to promote patient-centred care. This research aimed to develop a conceptual model to improve patient-centred T2DM management in clinical settings. A model is needed alongside robust approaches and context-driven research to aid surveillance, prevention, early diagnosis and patient-focused T2DM management (World Health Organization, 2016).

## Methods

This study sits with pragmatism. We adopted the constant comparison as a method (Charmaz, 2008) and not as a methodology as propounded by grounded theory (Glaser and Strauss, 1967). The grounded theory method was combined with the framework method (Ritchie and Spencer, 2002), which we adopted to gather rich and robust data through an iterative process, while the latter facilitated a transparent and systematic data analysis as advised by Merriam (2009). Interviews commenced after obtaining ethical approval from the university of the last author, and the Lagos State Ministry of Health Ethics Committee (LREC /06/10/682). All participants received a participant information sheet and gave informed consent before participation.

### Sampling

We used a sampling frame that encompassed a list of all the public secondary and tertiary health facilities in Lagos State. Furthermore, we adopted two types of purposive sampling (Holloway and Galvin, 2016). First, we used the maximum variation sampling method when selecting the hospitals. We ensured that we chose at least one hospital from each of the five administrative regions within Lagos State. Second, using expert and typical case sampling methods, we approached participants who had met the inclusion criteria as being qualified for 1–30 years with various work experience, nursing qualifications and educational levels.

### Characteristics of the study population and research participants

We selected six hospitals from different urban, suburban and rural regions across Lagos State. We anonymised them as AO1, BO1, CO1, DO1, EO1 and FO1 for confidentiality. Participants were given an alphanumeric label of P1–P8. The label represents the eight grades of the nurses interviewed: nursing officer one (N01), nursing officer two (NO2), sister, principal nursing officer (PNO), matron, assistant chief nursing officers (ACNOs), chief nursing officer (CNO) and apex. In this study, we interviewed nurses of different grades.

N01 and N02 are nurses who have practised for between one year and 10 years. Five nurses from this group participated. PNOs or matrons are more senior nurses. Four nurses from this group were interviewed. One of them had a bachelor’s degree qualification in nursing, and three had a diploma in nursing. Three ACNOs participated; one with BSc and master’s degrees, one with a BSc and one with a nursing diploma; one CNO with BSc and master’s degrees. Finally, we interviewed two apex nurses who are degree holders. Apex oversees the overall leadership and management of other nurses. In total, 17 nurses participated, with modal working experience of 18 years and median work experience of 15 years.

### Data collection

We collected data using face-to-face, in-depth semi-structured interviews. A pre-test interview guide was adopted. All participants had several years of work experience and nursing qualifications and were involved in both operational and strategic levels of patient management in public hospitals across Lagos, Nigeria. We engaged with participants to elicit their views for an in-depth understanding of factors to optimise patient-centred T2DM management. We posed three research questions.

- What factors threaten patient-centred T2DM management in clinical settings?
- How do patients with T2DM react to these factors?
- What approaches can inform person-centred T2DM management at a low or no cost?

**Figure 1.**
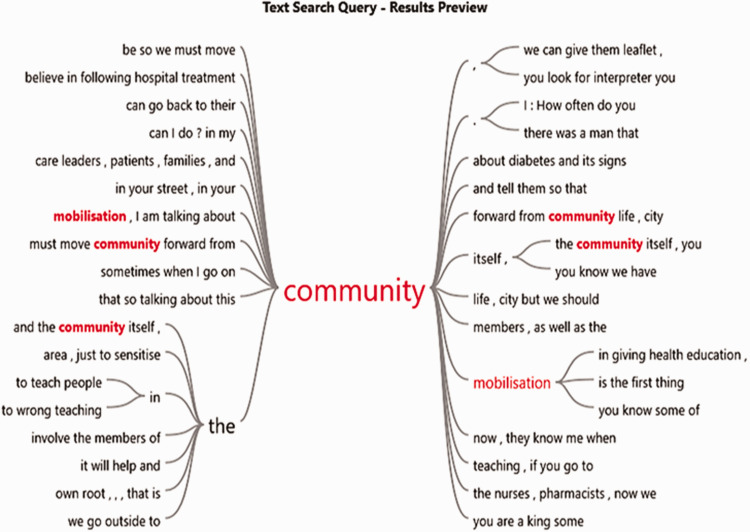
‘Community mobilisation’ as used in context (NVivo extract).

Each interview was audiotaped, lasted between 45 and 60 minutes and was transcribed verbatim. Throughout the data collection phase, constant comparison ensured the collection of robust data from the nurses through an iterative process. During this process, emerging information from all participants was logically integrated into the interview questions for a deeper exploration of the emerging themes (please see Figure 1 for an NVivo extract). We also adhered to data sufficiency as recommended by Bryman (2012) to ensure that adequate data were obtained to address the research aim. Data collection concluded at the point at which no new information, relevant to the research, was emerging from the participants.

### Data analysis

As the study aimed to develop a conceptual model to promote patient-centred T2DM care in clinical settings, we utilised the framework approach (Ritchie and Spencer, 2002) for a transparent and systematic analysis. During the five phases of data analysis: familiarisation; identifying a thematic framework; indexing; charting; and mapping and interpretation, we adopted both manual and NVivo analysis techniques, as recommended by Gale et al. (2013). Themes emerged from the data through a logical, rigorous, formal review and iteration of the data (Ritchie et al., 2013).

## Results

We interviewed a total of 17 practice nurses in six public hospitals across urban, suburban and rural regions of Lagos, Nigeria. Participant responses were analysed and are presented according to the three research questions we posed.

- What factors threaten patient-centred T2DM management in clinical settings?
- How do patients with T2DM react to these factors?
- What approaches can inform person-centred T2DM management at a low or no cost?

### Question one: What factors threaten patient-centred T2DM management in clinical settings?

We asked participants to discuss the factors that they felt threatened patient-centred T2DM management in the clinical settings in which they were working. We elicited from them elements which we interpreted to be unrealistic collaboration, rigid flexibility, uncomfortable approach and unsteady practice. Participants reported that the factors resulted when the organisational policies and decisions did not anchor on holistic patient-centredness.

#### Unrealistic collaboration

Nurses recounted that not all collaborations between health providers and volunteers, the government and international bodies, patients and traditional leaders, as well as hospitals and media, focused on patient-centred T2DM care. For instance

Some patients are being misled by their pastors or traditional leaders. There was a man that came in he was having a wound on his leg, so they said somebody came in and said that it was the witches and wizards, so he went to a pastor, and the pastor asked him to be burning something and be putting it on the wound for it to heal. P1 (N02)

A collaborative, patient-centred approach is empowering for patients to self-manage their T2DM. A patient-centred collaboration in healthcare is that which supports meaningful partnerships and collaboration with volunteers, international bodies, patients, traditional leaders and media agencies.

#### Rigid flexibility

Some clinical policies and decisions that aimed at flexible practices were reported by nurses as vague and rigid to achieve in real-world settings. For example, when patients are told to come to clinics for free blood glucose tests and no flexible payment arrangement is in place for them to buy their prescribed medications.

If they (the patients) cannot afford the drugs, we discuss among ourselves and refer the case to the social worker who will take over, but then most hospitals in Nigeria demand upfront payment before treatment meaning there is no flexible billing method. P3 (sister)

When a patient experienced this form of ‘rigid flexibility’, they refused professionals’ advice. The nurses felt that for a policy or decision to be considered flexible, it should be empowering and equity focused. Therefore, the participants suggested that a database needs to be instituted to record and evaluateT2DM cases treated across public hospitals in Nigeria. Participants also discussed the need for a connection between patients, the government, community members and professional bodies to make clinical approaches patient-focused and patient-driven to boost patient compliance.

The patients will then not listen to the health professionals; most of them just run away from the hospital and will come back with a diabetic foot. P4 (PN0)

I will just like to say that on the aspect of dietary [advice],… patient[s] do not really comply. Maybe because they are being forced by either their family members or health professionals to avoid some food. P1 (N02)

#### Uncomfortable approach

The participants expressed their concerns for uncomfortable operational approaches that existed in clinical practice in terms of the scare tactics and educational approaches adopted when counselling patients. An example of an uncomfortable approach is when a health professional uses health education approaches that increase patients’ fears.

By the time most of them are told the story of DM complications, they are always afraid but eager to learn. P1 (N02)

Participants explained that some health professionals found it challenging to communicate effectively, especially with uneducated patients. For instance, advising them to taste their urine for sweetness can be very disturbing for the patients.

Then you will also tell those stack illiterates, when you urinate, try to taste it, it must not taste like you are taking sugar. P4 (PN0)

The patient often felt uncomfortable with some of the strategies used by some health professionals. Some strategies adopted during health education of patients often worsened patients’ fears and worries about their condition.

and some health professional over-scrutinise the patients and screen them about what to eat or take. P3 (sister)

#### Inconsistent practice

The unsteady practice emerged from the words the participants used to describe inconsistent practices experienced by patients, especially during DM clinic days. Unsteady practices occur at a strategic level. As recounted by the participants, when many patients turned up for clinic appointments, they struggled to have a seat and had no easy access to the essential facility such as functional toilets and toilet access for disabled patients.

We see up to 400 patients in a day, times, 300, 250 on their clinic day and the clinic is not originally structured to accommodate many people. P7 (CNO)

Also, there were reported workforce and diabetes specialist shortages, and these had led to patients spending up to 6 hours waiting when they attended their clinical appointments. There were no consistent monitoring strategies or guidelines to guide practice around how patients were fed and monitored on the wards.

There are no diabetes specialist nurses in the department, and there is a shortage of manpower. P6 (ACNO)

We provide food for the patient when on admission. But well at the same time, you know? People still bring in food. For patients in the hospital, for showing that they care and things like that and they sneak in things. I will just like to say that on the aspect of dietary [advice], patients do not really comply. Maybe because they don’t really understand or maybe people, I don’t know, because patients still eat apart from the food given them. P7 (CNO)

### Question two: How do patients with T2DM react to these factors?

When participants were asked about patients’ reactions to the above-discussed factors, various burdens were expressed. The burdens described by the participants are linked with the rigid flexibility that results when a patient is advised to come to the hospital for testing and had no money or insurance to pay for their hypoglycaemic medications, as well as the unrealistic collaboration that exists. For instance, between the patients and their religious leaders in the rural areas, usually, some pastors who told individuals that DM was caused by witches or wizards.

The patients become so scared. P6 (ACNO)

Most of the patients believe that the witches and wizard cause it. P2 (N01)

Yes, out-of-pocket affect the patients because for the patients to come to you, so you have to relieve them of the financial burden so that they will be able to cooperate and when you tell them to come, they will come. DM drugs are expensive so that it will affect the health situation of Nigeria, yes it will. P8 (apex)

### Question three: What approaches can inform person-centred T2DM management at a low or no cost?

The participants considered T2DM management strategies that focused on low or no cost as patient-empowering and person-centred. In this section, we discuss the approaches highlighted by participants. Participants’ views are presented under four themes: empowering collaboration; empowering flexibility; empowering approach; and empowering practice.

#### Empowering collaboration

Nurses recounted that collaboration and partnership need to be driven by patient-centred intentions and not financial gains. The participants narrated that patients felt that collaboration and partnership should be tailored towards patients and their convalescence. However, effective media and platforms must be used to make collaboration empowering for the patients.

Use public address system be it through radio, through every form of… of which you can give the patients information. P5 (matron)

You know some of these elites; they have in-depth knowledge of the disease conditions, so we can also involve them to educate the patients. P5 (matron)

**Figure 2.**
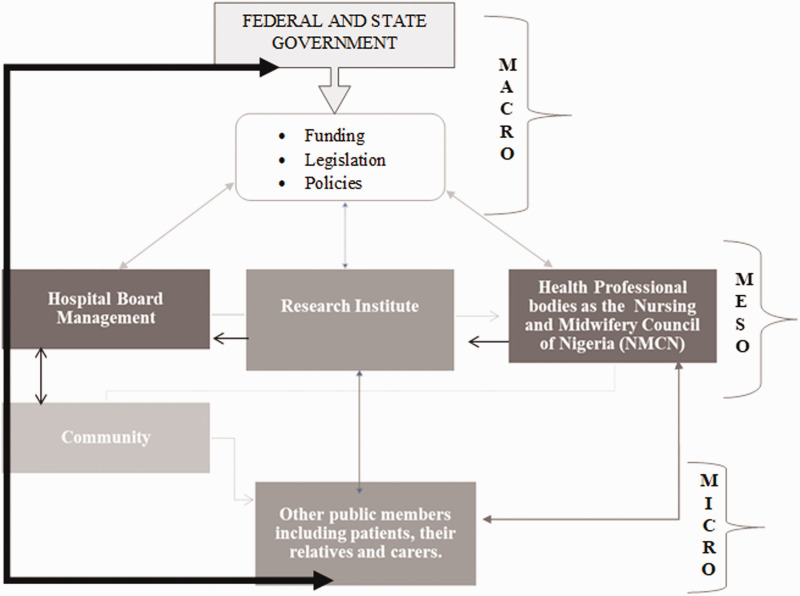
A conceptual model for patient-centred type 2 diabetes mellitus management in Nigeria.

The participants reported that only empowering collaboration could provide effective platforms for patients to become more knowledgeable about their DM and self-care to thrive. Such platforms would depend on timely, transparent and accurate cooperation among all key stakeholders. The government, non-governmental organisations (NGOs), patients’ relatives, health professionals, professional organisations, research organisations, religious and community leaders should be educated to educate patients when necessary. Please see Figure 2- A conceptual model for patient-centred type 2 diabetes mellitus management in Nigeria.

The first thing to do is mobilisation, and when I am talking about mobilisation, I am not just talking about ordinary mobilisation, I am talking about community mobilisation. P8 (apex)

community enlightenment through outreach programmes. P8 (apex)

#### Empowering flexibility

The participants acknowledged that flexibility is crucial to patient-centred care, but more importantly pointed out the benefit of empowering flexibility in health service delivery to patients with T2DM. Nurses stressed the empowering benefits of flexible clinic appointments, flexible follow-up care and flexible communication to patient-centredness. Also, nurses felt that flexible health financing would support patient-centredness in care in clinical settings. The link between health financing and health service delivery for patients diagnosed with T2DM was illuminated. The purpose of flexible health financing is to make funding available, as well as set financial incentives for the hospitals, such that individuals diagnosed with T2DM can flexibly access healthcare services and flexibly pay for their hypoglycaemic medicines.

support patients who are poor to have free treatment or flexible payment P8 (apex)

funding to make special pharmacy arrangement possible P1 (NO2)

#### Empowering approach and practice

The participants discussed the need to review clinical approaches to T2DM management in clinical settings frequently. Health professionals should uptake counselling approaches that can uphold patient empowerment, alleviate patients’ fears, correct their misconceptions about the causes of T2DM and heighten patients’ ability to self-manage their condition with less dependency.

most of them believe that DM is caused by the witches and wizard, so I have to tell them that their lifestyles matter. P6 (ACNO)

The nurses we interviewed gave insights on how DM education needs to be objective, evidence-based, informative and be provided concerning patients’ and their relatives’ decisions. Nurses suggested that all health stakeholders should, ‘say it as it is’ and ‘just do it right’. This suggests that health professionals should be honest and open when educating the patients about DM and to talk less and listen more to patients, reduce waiting time in clinics, provide more resources and training for staff, and ensure that DM specialists are available and accessible.

counsel them again one on one, so privacy and confidentiality are very important and so that one we will be able to gain their confident. P2 (N01)

Give patients flyers and leaflets to educate them. P3 (sister)

Tell patients the truth about their condition. P8 (apex)

even when the patients are in the hospital, we allow their relations, and we encourage the care of their relations. We tell their relations to also assist in caring for them. P6 (ACNO)

As explained by the research participants, listening to patients’ views is essential in clinical practice. However, while clinicians must listen more than they talk to patients, nurses stated that sometimes it might be vital to be firm with patients, especially when educating them on diet modification as a way of promoting their compliance. As well as encouraging patient networking for guidance and advice on lifestyle modification, nurses felt that patients who struggled to modify their diets benefitted from a ‘firm’ approach. However, while being firm, the participants suggested the use of jokes could improve effective communication and inform the development of a nurse–patient therapeutic relationship.

Crack jokes; get them in the mood and be specific and be precise not boring then let them participate so they will never be bored. P7 (CNO)

The hospital is trying. Here they have a club to promote patient networking. P5 (matron)

that is when caring for the patients with type 2 diabetes? OK, when you do one on one discussion with them, they try to be nice you explain in the language that they will understand what you are saying so if he is a Yoruba person, you speak Yoruba to them. P3 (sister)

### A conceptual model developed from the Nigerian context for patient-centred T2DM management

A conceptual model was logically developed from the data obtained from the nurses. We developed a model from the themes that emerged from the analysis of the interviews. The nurses who participated in this research objected to the idea that a standalone, macro-level factor could promote patient-centred T2DM care in clinical settings. Instead, the participants acknowledged core integration at macro, meso and micro levels was a critical element to patient-focused T2DM management.

At the macro-level (see Figure 2), the federal government should provide funds to subsidise hypoglycaemic medications for patients who require them. Furthermore, participants felt that lawmakers should formulate legislation and policies to advocate for patients and coordinate the delivery of valued and quality care to patients. The legislation and policies should be clear, concise and easily accessible and understandable for the people living with T2DM. At macro level, the Federal Ministry of Health should collaborate with frontline clinical staff and seek patients’ inputs regularly in order to formulate new policies and review existing policies for efficiency in light of how clinical practice and approaches can be flexible and empowering for the patients.

Through integration, the macro-level activities should link with the meso: for instance, the hospital board, research institutes and professional bodies to gain empowering collaboration, clinical approach, clinical practice and flexibility to patient care. As nurses in Nigeria recognise, this form of integration is crucial to abolishing unrealistic collaboration, unsteady practice, uncomfortable approaches and rigid flexibility which are detrimental to patient-centred T2DM management. The model shows that a structured and extended network among the government, community, professional bodies, research institutes, members of the public, religious leaders as well as patients and their relations are required to enable coordination and provision of patient-centred care to individuals diagnosed with T2DM. The community sits at the micro level of the model. It encompasses international stakeholders, NGOs, religious leaders, National Insurance Health Scheme providers, community leaders, food and drink companies and advocates, while the media, schools, family members, churches and mosques sit at the core centre of the micro level.

At the micro level, the nurses recognised the usefulness of community mobilisation by giving DM flyers to the people for community enlightenment and effective communication. Having DM specialists within the community to promote patient networking, social welfare, early identification of DM complications as well as providing free tests are models found productive in light of the promotion of patient-centred care to individuals living with T2DM. In addition, subsidised health services, outreach programmes, sharing experiences, timely regimen and group clinic education to patients were recounted as useful strategies to ameliorate patient health and DM outcomes.

To achieve a successful outcome in patient management, health providers who are involved in patient education should focus on health-promoting actions that will encourage patients to achieve realistic DM goals and DM outcomes (Corser et al., 2007; Utz et al., 2008). Partnership and collaboration are key elements of a successful strategy (International Diabetes Federation, 2017). Patients can collaborate to share their self-management experiences and discuss their regime and any associated challenges with their family members and friends. By doing this, the patients will be able to receive support from their loved ones. In the same way, health sectors and government can partner and collaborate at national and international levels to improve the effectiveness of T2DM management (World Health Organization, 2016).

Finally, it is paramount that integration is considered as a landmark, collaborative and empowering pathway for the optimisation of patient-centred T2DM management. The macro, meso and micro levels should make a collective alliance to heightening DM care and support to the individuals living with T2DM.

## Discussion

This research sought nurses’ contextual perceptions of clinical practices and operations in light of T2DM management across public hospitals in Lagos, Nigeria, for the development of a conceptual model to optimise patient-centred T2DM care in clinical settings.

### Collaboration

The findings of this research support that collaboration is a typical element promoting T2DM management in the clinical setting. Factors such as blame, bureaucracy and mistrust can jeopardise collaboration (McSherry, 2010). The outcome of our research adds that it takes empowering collaboration to heighten patient-centred T2DM management for optimal health outcomes of patients.

Patient-centred care in DM management can be achieved through collaboration (Inzucchi et al., 2012). Katon et al. (2010) found that patient-centred collaboration promoted patient satisfaction for people with diabetes and improved their quality of life. Collaboration curbed depression among low-income individuals with diabetes (Ell et al., 2010). Trief et al. (2011) found that timely telephone intervention promoted collaboration for the patient with T2DM and their partners. Similarly, Lyles et al. (2011) found that mobile phones are useful tools for a web-based collaborative care programme for T2DM. Lyles et al. (2011) showed that benchmarking is a product of a collaboration that has led to improved quality of care in T2DM. At the same time, when patients interact with each other to learn lifestyle-modifying strategies through collaboration, it had a significant impact on improving the glycaemic levels among patients with T2DM (Hermans et al., 2013; Parkinson et al., 2016).

The findings of Qi et al. (2015) aligned with what the participants of this research considered as patient networking, a vital component of collaboration for patient-centred care. Several approaches can support patient-centred partnership; however, there is a factor that sums it all up – having a culturally sensitive healthcare delivery system. When healthcare delivery is designed to be culturally appropriate, there is a significant improvement in self-reported diabetes to community health (Spencer et al., 2011). The outcome of our research affirms that integrated care is a suitable approach to promote patient-centred T2DM management as supported by Nuño et al. (2012). Zhang et al. (2015) concur the outcome of our research by demonstrating that patients with diabetes, receiving an integrated model of care, had a reduction in the number of hospitalisations. The findings of the current research align with critical areas of care organisation for patients with T2DM as recommended by the International Diabetes Federation (2017).

### Clinical practice

As highlighted in these research findings, clinical practices that target successful diabetes self-management education (DSME) are essential to promoting patient-centred T2DM management. However, the outcome of our research suggests that empowering clinical practice can optimise DSME. DSME is an innovative approach that helps patients to be independent and self-involved in their care, thus improving patient outcomes. It is crucial to communicate firmly and cautiously with patients because while being firm, poor communication due to linguistic challenges may result in undue tension between clinicians and patients (Martin, 2014).

To improve practice, Ismail-Beigi (2012) supports that clinical decisions should consider cost, side effects of medicines and long-term safety and effects of therapeutic agents on patient management. As suggested in this study, health professionals should address issues relating to patient management in a timely fashion. Timely communication is crucial to reducing treatment delays among physicians (Christie and Channon, 2014; Strain et al., 2014). In our research, the nurses added that patients should be allowed to talk more while the clinician interviewing should listen more and talk less, bearing in mind patient-centredness. Clinicians need to choose their words and language carefully (Sogg et al., 2018).

### Flexible approach and practice

The research outcome made clear that operational approaches and strategic practices to patient care need to be flexible in many ways which include having adequate staff to care for patients, flexible payment options and flexible counselling strategies. Nigeria has one of the highest out-of-pocket payment plans in the world. About 70% of patients pay for their health directly, and 30% pay for their health through tax-based revenue, donor funding, social health insurance, community-based health insurance and private health insurance (Uzochukwu et al., 2015). The World Health Organization (2016) reiterates health financing as a critical determinant for achieving universal health coverage.

Most public and private hospitals in Nigeria demand upfront payment before they can treat patients (Aregbesola and Khan, 2018). Around 4% of households spend more than half of their total household expenditure on healthcare, and 12% spend more than a quarter. The consequences of this health financing to the management of a long-term condition such as T2DM are substantial, and it can aggravate the poverty of many patients living with T2DM in Nigeria. As revealed by this research, the hospital billing method for patients needs to be flexible and not rigidly flexible for patients to become more compliant with their regimen. It is also imperative to ensure that flexible dietary advice and a subsidised form of pharmaceutical care are provided for people who require it.

In the United Kingdom (UK), for instance, guidelines for the dietary management of patients with T2DM have been modified to accommodate a more flexible approach to weight loss and individualised approaches to patient management (Dyson et al., 2011). Patients with T2DM are educated on the national recommendations for patients with DM who preferred alcohol (Dyson et al., 2018).

Various clinical approaches for patient-centred T2DM management exist. As suggested by the research results, providing useful DM information to patients is a valuable idea for promoting patient-centred T2DM management. However, to do this efficiently, especially in rural areas, telehealth, a technological approach to health education, will be a great asset. Telehealth has proved useful in promoting patient access to DM health education. Nurses and dietitians successfully conducted a one-year remote DSME to an ethnically diverse, rural and underserved population in rural South Carolina. Patients managed through telehealth and had improved metabolic outcomes and reduced cardiovascular complications (Davies et al., 2010). Web-based behavioural interventions integrated with e-research strategies led to favourable outcomes for patients with T2DM (Ramadas et al., 2011).

A patient empowerment programme (PEP) is effective in improving the clinical outcomes and reducing the general outpatient clinic utilisation rate over 12 months (Wong et al., 2014). Empowering T2DM patients on the self-management of their disease can enhance the quality of diabetes care in primary care on clinical outcomes and health service utilisation rates in patients with T2DM, in the primary care setting. It is essential, therefore, that healthcare professionals continue to advocate collaboration and flexibility, as found in this research; also, promoting patient-centred treatment options for the optimisation of patient outcomes in Nigeria (Bosun-Arije et al., 2017). In addition, it is cogent that PEP desists from unpleasant activities that can potentially jeopardise the PEP objectives.

### Limitations

None of the participants was a diabetes nurse specialist, from whom a more theory-driven insight could have emerged. As nurses are key clinicians in healthcare settings, other healthcare professionals such as doctors and laboratory scientists could have contributed to the model. If they were included, their views would have contributed to the robustness of the data that informed the model proposed. Patient perspectives would also have added to the data that informed the model. We plan to address these limitations in future research.

## Conclusions

Patient-centredness should be a priority in healthcare. The unique nurse-led model can serve as an asset to improve patient outcomes not only in countries with fragile healthcare systems but also in developed countries with flourishing healthcare services. In the context of clinical structure and culture in health settings, nurses are an indispensable part of the health profession and their experiences should continue to inform person-centred T2DM management. The outcome of this research proposes a conceptual model that can inform cost-effective person-centred T2DM management in clinical settings.

## Key points for policy, practice and/or research

- Collaboration, flexibility, clinical approach and clinical practice must anchor on empowerment to uphold patient-centred T2DM management and quality health services in clinical settings.
- Nurses’ insights informed an integrated model for the optimisation of patient-centred T2DM management in clinical settings.
- A nurse-led conceptual model can strengthen collaborative and flexible working for care optimisation of patients with T2DM in clinical practice.

## References

[bibr1-17449871211021137] Adeniyi AF Ogwumike OO Oguntola DA et al.>(2015) Interrelationship among physical activity, quality of life, clinical and sociodemographic characteristics in a sample of Nigerian patients with type 2 diabetes. African Journal of Physiotherapy and Rehabilitation Sciences 7(1–2): 12–18.

[bibr2-17449871211021137] Anakwue R Arodiwe E Ofoegbu E (2013) The prevalence and control of hypertension among patients with type 2 diabetes mellitus in Nigeria. Journal of College of Medicine 17(2): 11.

[bibr3-17449871211021137] Aregbeshola BS Khan SM (2018) Out-of-pocket payments, catastrophic health expenditure and poverty among households in Nigeria 2010. International Journal of Health Policy and Management 7(9): 798. 30316228 10.15171/ijhpm.2018.19 PMC6186489

[bibr4-17449871211021137] Arogundade FA (2013) Kidney transplantation in a low-resource setting: Nigeria experience. Kidney International Supplements 3(2): 241–245. 25018990 10.1038/kisup.2013.23 PMC4089648

[bibr5-17449871211021137] Bosun-Arije FS Ling J Graham Y et al.>(2017) Global insights into the clinical management of type 2 diabetes: a context-specific view from Nigeria on drivers and barriers to clinical nursing management. Diabetic Medicine 34(Suppl. 1): 177.

[bibr6-17449871211021137] Bosun-Arije FS Ling J Graham Y et al.>(2019) A systematic review of factors influencing type 2 diabetes mellitus management in Nigerian public hospitals. International Journal of Africa Nursing Sciences 11: 100151.

[bibr7-17449871211021137] Bosun-Arije FS, Ling J, Graham Y, et al. (2020) Organisational factors influencing non-pharmacological management of type 2 diabetes mellitus (T2DM) in public hospitals across Lagos, Nigeria: A qualitative study of nurses' perspectives. *Diabetes research and clinical practice* 166: p.108288. 10.1016/j.diabres.2020.108288 32615277

[bibr10-17449871211021137] Bryman A (2012) Sampling in qualitative research. Social Research Methods 4: 415–429. Oxford University Press.

[bibr11-17449871211021137] Charmaz K (2008) Reconstructing grounded theory. In: *The Sage handbook of social research methods*, Sage, pp. 461–478.

[bibr12-17449871211021137] Chijioke A Adamu AM Makusidi AM (2010) Mortality patterns among type 2 diabetes mellitus patients in Ilorin, Nigeria. Journal of Endocrinology, Metabolism and Diabetes of South Africa 15(2): 79–82.

[bibr13-17449871211021137] Chinenye S Uloko AE Ogbera AO et al.>(2012) Profile of Nigerians with diabetes mellitus – Diabcare Nigeria study group (2008): results of a multicenter study. Indian Journal of Endocrinology and Metabolism 16(4): 558. 22837916 10.4103/2230-8210.98011 PMC3401756

[bibr14-17449871211021137] Christie D Channon S (2014) The potential for motivational interviewing to improve outcomes in the management of diabetes and obesity in paediatric and adult populations: a clinical review. Diabetes, Obesity and Metabolism 16(5): 381–387. 10.1111/dom.12195 PMC4237607 23927612

[bibr15-17449871211021137] Corser W Holmes-Rovner M Lein C et al.>(2007) A shared decision-making primary care intervention for type 2 diabetes. The Diabetes Educator 33(4): 700–708. 17684171 10.1177/0145721707304086

[bibr16-17449871211021137] Davis RM Hitch AD Salaam MM et al.>(2010) TeleHealth improves diabetes self-management in an underserved community: diabetes TeleCare. Diabetes Care 33(8): 1712–1717. 20484125 10.2337/dc09-1919 PMC2909047

[bibr18-17449871211021137] Dyson PA Kelly T Deakin T et al.>(2011) Diabetes UK evidence-based nutrition guidelines for the prevention and management of diabetes. Diabetic Medicine 28(11): 1282–1288. 21699560 10.1111/j.1464-5491.2011.03371.x

[bibr19-17449871211021137] Dyson PA Twenefour D Breen C et al.>(2018) Diabetes UK evidence-based nutrition guidelines for the prevention and management of diabetes. Diabetic Medicine 35(5): 541–547. 29443421 10.1111/dme.13603

[bibr20-17449871211021137] Ell K Katon W Xie B et al.>(2010) Collaborative care management of major depression among low-income, predominantly Hispanic subjects with diabetes: a randomised controlled trial. Diabetes Care 33(4): 706–713. 20097780 10.2337/dc09-1711 PMC2845010

[bibr21-17449871211021137] Fasanmade OA Odeniyi IA Amira CO et al.>(2013) Association of body mass index and abdominal adiposity with atherogenic lipid profile in Nigerians with type 2 diabetes and/or hypertension. Nigerian Medical Journal: Journal of the Nigeria Medical Association 54(6): 402. 24665155 10.4103/0300-1652.126296 PMC3948963

[bibr22-17449871211021137] Gale NK Heath G Cameron E et al.>(2013) Using the framework method for the analysis of qualitative data in multi-disciplinary health research. BMC Medical Research Methodology 13(1): 1–8. 24047204 10.1186/1471-2288-13-117 PMC3848812

[bibr23-17449871211021137] Glaser BG Strauss AL (1967) The discovery of grounded theory, London: Weidenfield and Nicolson.

[bibr24-17449871211021137] Hermans MP Elisaf M Michel G et al.>(2013) Benchmarking is associated with improved quality of care in type 2 diabetes: the OPTIMISE randomised, controlled trial. Diabetes Care 36(11): 3388–3395. 23846810 10.2337/dc12-1853 PMC3816864

[bibr25-17449871211021137] Holloway I Galvin K (2016) Qualitative Research in Nursing and Healthcare, Hoboken: John Wiley and Sons, Inc.

[bibr26-17449871211021137] International Diabetes Federation (2014) *Regional Fact sheet*. Available at: www.idf.org (accessed February 2018).

[bibr27-17449871211021137] International Diabetes Federation (2015) Available at: https://www.diabetesatlas.org/upload/resources/previous/files/7/IDF%20Diabetes%20Atlas%207th.pdf (accessed October 11, 2021).

[bibr28-17449871211021137] International Diabetes Federation (2017) Diabetes Atlas, 8th edition, IDF. https://diabetesatlas.org/upload/resources/previous/files/8/IDF_DA_8e-EN-final.pdf (accessed October 11, 2021).

[bibr29-17449871211021137] Inzucchi SE Bergenstal RM Buse JB et al.>(2012) Management of hyperglycaemia in type 2 diabetes: a patient-centred approach. Position statement of the American Diabetes Association (ADA) and the European Association for the Study of Diabetes (EASD). Diabetologia 55(6 1577–1596. 22526604 10.1007/s00125-012-2534-0

[bibr30-17449871211021137] Ismail-Beigi F (2012) Glycemic management of type 2 diabetes mellitus. New England Journal of Medicine 366(14): 1319–1327. 10.1056/NEJMcp1013127 22475595

[bibr31-17449871211021137] Iwuala SO Olamoyegun MA Sabir AA et al.>(2015) The relationship between self-monitoring of blood glucose and glycaemic control among patients attending an urban diabetes clinic in Nigeria. Annals of African Medicine 14(4): 182. 26470743 10.4103/1596-3519.155992

[bibr32-17449871211021137] Katon WJ Lin EH Von Korff M et al.>(2010) Collaborative care for patients with depression and chronic illnesses. New England Journal of Medicine 363(27): 2611–2620. 10.1056/NEJMoa1003955 PMC3312811 21190455

[bibr35-17449871211021137] Lyles CR Harris LT Le T et al.>(2011) Qualitative evaluation of a mobile phone and web-based collaborative care intervention for patients with type 2 diabetes. Diabetes Technology and Therapeutics 13(5): 563–569. 21406018 10.1089/dia.2010.0200

[bibr36-17449871211021137] McSherry R and Warr J (2010) Factors influencing collaborative working. Implementing Excellence In: *Your Health Care Organization: Managing, Leading And Collaborating: managing, leading and collaborating*, Jul 1:97.

[bibr37-17449871211021137] Martin G (2014) “Well I don’t know what to say to that”: Exploring tensions between the voices of Medicine and the lifeworld in the management of diabetes: the case of an immigrant patient. European Journal of Applied Linguistics 2(2): 204–232.

[bibr38-17449871211021137] Merriam SB (2009) *Qualitative Research: a Guide to Design and Implementation* (rev. and exp. ed.). San Francisco,: Jossey-Bass.

[bibr39-17449871211021137] National Bureau of Statistics (2015) *Poverty in Nigeria*. Available at: https://www.nigerianstat.gov.ng/ (accessed June 2018).

[bibr40-17449871211021137] Nuño R Coleman K Bengoa R et al.>(2012) Integrated care for chronic conditions: the contribution of the ICCC Framework. Health Policy 105(1): 55–64. 22071454 10.1016/j.healthpol.2011.10.006

[bibr41-17449871211021137] Olamoyegun M Ibraheem W Iwuala S et al.>(2015) Burden and pattern of microvascular complications in type 2 diabetes in a tertiary health institution in Nigeria. African Health Sciences 15(4): 1136–1141. 26958014 10.4314/ahs.v15i4.12 PMC4765393

[bibr42-17449871211021137] Onakpoya OH Adeoye AO Kolawole BA (2010) Determinants of previous dilated eye examination among people with type II diabetes in Southwestern Nigeria. European Journal of Internal Medicine 21(3): 176–179. 20493418 10.1016/j.ejim.2010.01.009

[bibr43-17449871211021137] Parkinson S Eatough V Holmes J et al.>(2016) Framework analysis: a worked example of a study exploring young people’s experiences of depression. Qualitative Research in Psychology 13(2): 109–129.

[bibr44-17449871211021137] Qi L Liu Q Qi X et al.>(2015) Effectiveness of peer support for improving glycaemic control in patients with type 2 diabetes: a meta-analysis of randomised controlled trials. BMC Public Health 15(1): 471. 25943398 10.1186/s12889-015-1798-y PMC4425885

[bibr45-17449871211021137] Ramadas A Quek KF Chan CKY et al.>(2011) Web-based interventions for the management of type 2 diabetes mellitus: a systematic review of recent evidence. International Journal of Medical Informatics 80: 389–405. 21481632 10.1016/j.ijmedinf.2011.02.002

[bibr46-17449871211021137] Ritchie J and Spencer L (2002) *Qualitative data analysis for applied policy research*. In: Analyzing qualitative data (pp. 187-208). Routledge.

[bibr47-17449871211021137] Ritchie J, Lewis J, Nicholls CMC, et al. (eds) (2013) *Qualitative Research Practises. A Guide for Social Science Students and Researchers*. Sage.

[bibr48-17449871211021137] Sogg S Grupski A Dixon JB (2018) Bad words: why language counts in our work with bariatric patients. Surgery for Obesity and Related Diseases 14(5): 682–692. 29525262 10.1016/j.soard.2018.01.013

[bibr49-17449871211021137] Spencer MS Rosland AM Kieffer EC et al.>(2011) Effectiveness of a community health worker intervention among African American and Latino adults with type 2 diabetes: a randomised controlled trial. American Journal of Public Health 101(12): 2253–2260. 21680932 10.2105/AJPH.2010.300106 PMC3222418

[bibr51-17449871211021137] Strain WD Cos X Hirst M et al.>(2014) Time to do more: addressing clinical inertia in the management of type 2 diabetes mellitus. Diabetes Research and Clinical Practice 105(3): 302–312. 24956964 10.1016/j.diabres.2014.05.005

[bibr52-17449871211021137] Trief P Sandberg JG Ploutz-Snyder R et al.>(2011) Promoting couples collaboration in type 2 diabetes: the diabetes support project pilot data. Families, Systems, and Health 29(3): 253. 10.1037/a0024564 21744962

[bibr53-17449871211021137] Utz SW Williams IC Jones R et al.>(2008) Culturally tailored intervention for rural African Americans with type 2 diabetes. The Diabetes Educator 34(5): 854–865. 18832290 10.1177/0145721708323642 PMC3622474

[bibr54-17449871211021137] Uzochukwu BSC Ughasoro MD Etiaba E et al.>(2015) Health care financing in Nigeria: implications for achieving universal health coverage. Nigerian Journal of Clinical Practice 18(4): 437–444. 25966712 10.4103/1119-3077.154196

[bibr56-17449871211021137] Wong CK Wong WC Lam CL et al.>(2014) Effects of Patient Empowerment Programme (PEP) on clinical outcomes and health service utilisation in type 2 diabetes mellitus in primary care: an observational matched cohort study. PloS One 9(5): e95328. 24788804 10.1371/journal.pone.0095328 PMC4006782

[bibr57-17449871211021137] World Bank (2017) *Current health expenditure (% of GDP) – Sub-Saharan Africa, World*. Available at: https://data.worldbank.org/indicator/SH.XPD.CHEX.GD.ZS?locations=ZG-1W (accessed June 2020).

[bibr590-17449871211021137] World Health Organization (2016). Global report on Diabetes. Available at: http://docs.dpaq.de/10605-diabetes_who_embargoed-who-global-report-on-diabetes.pdf (accessed May 2016).

[bibr61-17449871211021137] Zhang J Donald M Baxter KA et al.>(2015) Impact of an integrated model of care on potentially preventable hospitalisations for people with type 2 diabetes mellitus. Diabetic Medicine 32(7): 872–880. 25615800 10.1111/dme.12705

